# The Effect of Vitamin C (Ascorbic Acid) in the Treatment of Patients with Cancer: A Systematic Review

**DOI:** 10.3390/nu11050977

**Published:** 2019-04-28

**Authors:** Gwendolyn N.Y. van Gorkom, Eline L. Lookermans, Catharina H.M.J. Van Elssen, Gerard M.J. Bos

**Affiliations:** Division of Hematology, Department of Internal Medicine, GROW-School for Oncology and Developmental Biology, Maastricht University Medical Center, 6229 HX Maastricht, The Netherlands; e.lookermans@student.maastrichtuniversity.nl (E.L.L.); janine.van.elssen@mumc.nl (C.H.M.J.v.E.); gerard.bos@mumc.nl (G.M.J.B.)

**Keywords:** vitamin C, ascorbic acid, cancer, supplementation, overall survival, adverse events

## Abstract

Many cancer patients on intensive chemotherapy lack vitamin C. Vitamin C stimulates the production and activation of immune cells, so perhaps supplementation could be used to improve the immunity in those patients. This review assesses the effectiveness and safety of vitamin C administration in cancer. The PubMed and EMBASE databases were searched and all study designs except for phase I studies, and case reports were included in this review. A total of 19 trials were included. In only 4 trials randomization was used to determine if patients received vitamin C or a placebo. The result of this review does not prove that there is a clinically relevant positive effect of vitamin C supplementation in cancer patients in general on the overall survival, clinical status, quality of life (QOL) and performance status (PS), since the quality of the studies published is low. Interventions and patient groups are very diverse, hence an effect in some patient groups is possible. There seems to be a better effect with intravenous than oral administration. Nevertheless, treatment with vitamin C is safe with minimal side effects. Thereby, we think it is safe to examine the effects of vitamin C on specific groups of patients in a randomized controlled setting.

## 1. Introduction

Vitamin C is an essential micronutrient, that plays an important role in numerous physiological processes in the human body. Unlike most mammals, humans lack the ability to generate endogenous vitamin C due to a mutation in the *GULO* gene and are thereby completely dependent on dietary intake. The biological efficacy of vitamin C depends on its redox abilities and it functions as a cofactor in many enzymatic reactions. In physiological concentrations, it also functions as an antioxidant. 

By the 1970s, Nobel Price winner Linus Pauling had already developed a strategy to use intravenous (IV) vitamin C in cancer patients [1,2]. He treated patients with advanced cancer with high doses of vitamin C and reported a positive effect on survival. However, these studies have been methodologically criticized on several aspects such as data collection and data analysis. This resulted in a limited use of vitamin C in cancer patients. Other studies performed afterwards could also not reproduce these results; however, opposed to the intravenous use of vitamin C by Pauling et al, in most of these studies, oral vitamin C supplementation was used [3]. Pharmacokinetic studies show that the way of administration makes a big difference as peak plasma vitamin C concentrations after intravenous administration are much higher (up to 70-fold) than after oral intake [4]. Peak plasma concentrations also continue to increase when the intravenous dose of vitamin C is increased, while peak plasma concentrations plateau around 220 µM even though oral doses are increased.

There are multiple hypotheses about the way vitamin C has anti-tumor effects. An important possible mechanism of action is that in pharmacological concentrations (especially after intravenous use) vitamin C functions as a pro-oxidant and stimulates the formation of hydrogen peroxide. This hydrogen peroxide can create reactive oxygen species (ROS), that directly have cytotoxic activity on cancer cells [5]. Another important hypothesis is that vitamin C can create important epigenetic changes due to its effect on 2-oxoglutarate-dependent dioxygenases, like histone and DNA demethylases [6]. In preclinical studies investigators also show that vitamin C can have a synergetic effect with some types of chemo- and immunotherapy [7,8,9,10,11].

Additionally we showed in pre-clinical studies that vitamin C has an important role in the immune system, as it stimulates the production and/or activation of immune cells, like T-lymphocytes and natural killer cells, that have a function in fighting against pathogens and cancer cells [12,13,14].

In our previous research on vitamin C we noticed that many of our patients receiving intensive chemotherapy and/or stem cell transplantations for hematological malignancies have low vitamin C plasma concentrations [15]. This could be the result of low dietary intake of these patients or of an increased need for vitamin C in tumor cells or in immune cells. In extension of our results, other researchers observed that low vitamin C plasma levels in patients with various types of advanced cancer were associated with worse survival [16]. 

Patients that receive intensive chemotherapy and/or stem cell transplantations are prone for infectious complications. Boosting their immune system with vitamin C to hasten immune recovery and thereby prevent infectious complications is attractive, since vitamin C is cheap and generally available. However, since some vitamins have been shown to promote cancer development, we were interested in the effects of vitamin C on cancer progression and its safety. To this end, we conducted a systematic review of the literature on vitamin C administration in cancer patients. We focused on administration route, efficacy and on the side-effects in combination with or without other cancer treatment.

## 2. Materials and Methods 

### 2.1. Objectives

The aim of this review is to assess the effectiveness of vitamin C in the treatment of cancer, with or without adjuvant standard anti-cancer treatment like chemotherapy and radiotherapy. 

We researched the literature on the following hypotheses:

— Vitamin C administration is more effective in the treatment of cancer than placebo or no treatment in susceptible populations.
— Different routes of vitamin C administration (intravenous/oral) may differ in effectiveness in treating cancer.

The reached serum and/or tissue vitamin C concentrations with supplementation were also of interest and were noted when given.

An attempt was also made to quantify toxicity and side effects of vitamin C and the findings were considered in the discussion to determine the risk–benefit ratio of the treatment.

### 2.2. Protocol and Registration 

This systematic review was written conform the PRISMA statement for reporting systematic reviews of studies that evaluate health care interventions [17]. It is registered with the University of York Centre for Reviews and Dissemination International Prospective Register of Systematic Reviews.

### 2.3. Eligibility Criteria 

#### 2.3.1. Types of Studies

Studies on the effect of vitamin C administration in cancer patients after diagnosis were included. All study designs were allowed except for Phase I trials and case reports, since there was a lack of extensive randomized controlled trials (RCTs) but the quality of the studies was weighted during analysis and discussion. Language was restricted to English. 

#### 2.3.2. Types of Participant

**Inclusion criteria**

— Studies with patients of all ages and both genders with all types of diagnosed cancer.

**Exclusion criteria**

— Studies investigating the effect of nutritional supplements.

— Studies on the effect of vitamin C administration in the prevention of cancer. 

#### 2.3.3. Types of Intervention

Studies on the effect of clinical vitamin C administration, as mono-therapy or in combination with other standard cancer treatment regimes. The dose and mode of delivery were considered in subgroup analyses. 

#### 2.3.4. Types of Outcome Measures

Primary outcome measure was overall survival. Secondary outcome measures were progression-free survival, tumor response, response rate, disease-free survival, adverse effects, quality of life (QOL), clinical response and performance status (PS). 

### 2.4. Literature Search

Studies were identified by searching the PubMed and EMBASE databases and snowballing from review articles and relevant studies. The last search was run on the 11th of March 2019.

The following search terms were used to conduct the search: Neoplasms; Cancer; Malignancy; Leukemia; Lymphoma; Ascorbic acid; Vitamin C; Ascorbate; Dehydroascorbic acid; Randomized controlled trial; RCT; Randomized; Controlled clinical trial; Prospective study; Clinical trial; Case-control; Cohort; Phase 2; Observational study; Reduced infection; Overall survival; Progression-free survival; Toxicity; Quality of life; Tumor response; Response rate; Disease-free survival. The complete search strategy is shown in Supplementary File S1. Bibliographies of identified articles were also reviewed and searched manually for additional references. 

### 2.5. Data Collection and Analysis

Assessment of eligibility of the articles for inclusion in this review was performed and peer reviewed by two of the authors. The identified articles were screened on title and abstract in agreement with the inclusion and exclusion criteria by E. Lookermans (E.L.), who discarded studies that were clearly ineligible but aimed to be overtly inclusive rather than risk losing relevant studies. Subsequent full text assessment resulted in the final study selection. 

Data were collected by E. L. and peer reviewed by G. van Gorkom (G.G.) with use of a data extraction sheet based on the Cochrane Consumers and Communication Review Group’s data extraction template [18]. For each included study, information was extracted regarding the methods of the study (aim, study design, number of groups), the participants (number of patients, patient description, geographic location, methods of recruitment, inclusion criteria for participation, exclusion criteria for participation, age, gender) the intervention (vitamin C treatment, dose, schedule, mode of delivery, additional treatment, previous cancer treatments received, setting) and the outcomes and comparison groups (primary outcome measures, secondary outcome measures, method of assessing outcome measures, method of follow-up for non-respondents, outcome assessment, length of follow-up, frequency, relevant adverse events). Discrepancies in the data extraction were resolved through discussion. 

The quality of all eligible studies was assessed independently by two authors (E.L. and G.G.) with use of predefined risk of bias criteria, with discrepancies resolved by discussion and a third author (G.B.) when necessary. For randomized-controlled trials “The Cochrane Collaboration’s tool for assessing risk of bias in randomized trials” was used [19], for non-randomized comparison studies (studies were there is a group of patients included without vitamin C) the ROBINS-1 tool was used, a Cochrane risk of bias assessment tool for non-randomized studies of interventions [20] and the Effective Public Health Practice Project (EPHPP) Quality Assessment Instrument was used for non-comparison studies [21]. These tools were used to make judgments about the extent of bias that may be present in each of the studies and to rate the information in each component of the paper (Supplementary File S2). 

## 3. Results

### 3.1. Study Selection

**Figure 1:**
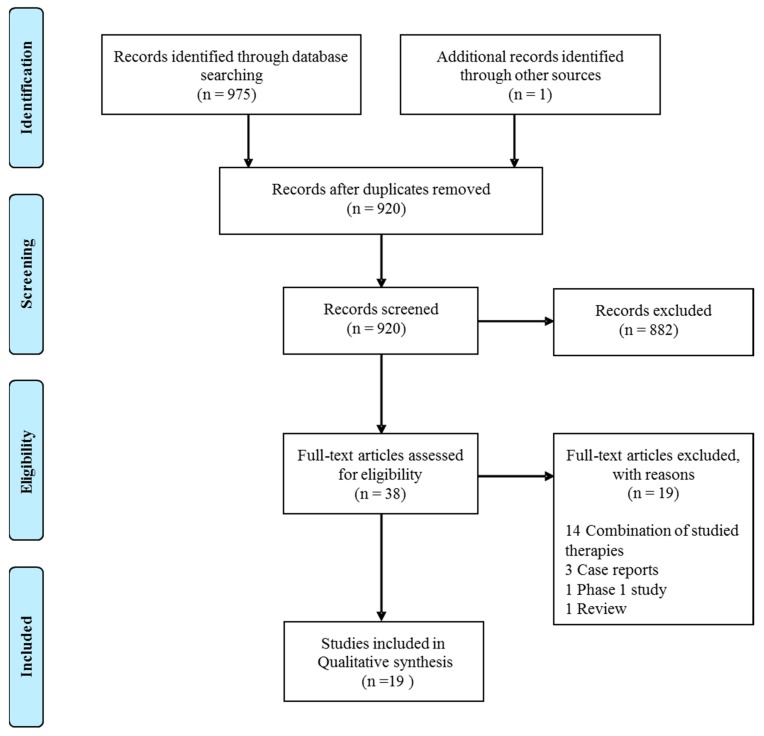
Flow diagram of the article selection.

A total of 975 articles was retrieved by the PubMed and EMBASE databases search. An additional article was found through article references, bringing the total number of records suitable for further evaluation to 976. After removal of duplicates there were 920 articles left for investigation. By scanning the title and abstract of these records, 882 records were excluded because they clearly did not match the inclusion criteria (mostly it were preclinical studies). Thirty-eight articles were evaluated on their full text. Of these articles, 19 records were excluded based on the inclusion criteria. In most of these articles, vitamin C supplementation was not the primary intervention, but combined with other experimental treatments, like other vitamins or arsenic trioxide. This resulted in 19 records being included for qualitative synthesis (Figure 1). 

### 3.2. Study Characteristics 

The study characteristics of the 19 articles selected for this review are described in Table 1. 

#### 3.2.1. Participants

The number of participants in these studies ranges from 14 to 1826. 

All studies described different individual patients except the 2 studies by the same authors. In both of these studies, 100 patients with terminal cancer which were treated with IVC were compared to 1000 similar control patients. The second study 10 of the original studied patients were replaced since there were not enough suitable control patients, but 90 patients and most of the controls were identical to the first study [1,23].

Most trials included patients with a variety of cancer types, and most of the time patients were in a terminal or advanced stage. In only 6 studies a specific cancer type was treated: 2 times breast cancer, 1 ovarian cancer, 1 colorectal cancer, 1 prostate cancer and 1 acute myeloid leukemia.

All trials included both sexes, except for the studies on breast cancer and ovarian cancer that logically treated only women and the study on prostate cancer that logically treated only men. The average age of the participants was approximately 60 years.

#### 3.2.2. Intervention 

In 8 studies, vitamin C was given intravenously (IV), all in different doses and time intervals [22,25,26,27,28,33,35,37]. In 8 studies intravenous vitamin C (IVC) was given followed by or in combination with oral vitamin C supplementation [1,2,23,24,30,31,34,36]. In 3 studies vitamin C supplementation was only prescribed orally [3,29,32]. 

#### 3.2.3. Other Treatments

In 7 studies, conventional anti-cancer treatment was given in addition to the administration of vitamin C [22,24,25,27,34,35,37]. In 6 studies, this was not specifically documented, but it seems unlikely that patients had concomitant treatments [3,26,28,30,32,36] and in 6 articles it was written that no additional treatment was given at the time of the intervention with vitamin C [1,2,23,29,31,33]. In 4 articles, the researchers described that patients received conventional cancer therapy prior to participation [2,31,32,35], 13 articles report no information of previous treatments [1,3,22,23,24,25,26,27,28,30,33,34,36] and in 2 studies patients received no prior treatment [29,37]. 

#### 3.2.4. Outcome Measures 

Ten articles discuss the effect of vitamin C on overall survival [1,3,23,24,26,27,29,30,32,37], 9 articles the effect on clinical response in general [2,25,26,28,29,30,31,33,37], 7 on QOL and PS [3,26,30,31,34,35,36] and 14 articles report on the safety and toxicity of vitamin C treatment [2,3,22,25,26,27,29,30,31,33,34,35,36,37]. 

### 3.3. Risk of Bias in Included Studies 

**Figure 2:**
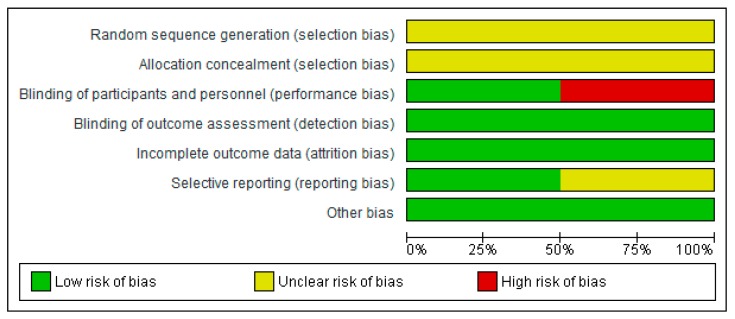
Itemized Judgments for risk of bias based on “The Cochrane Collaboration’s tool for assessing risk of bias in randomized trials”. Risk is presented percentages across all included RCTs.

Figure 2 presents the bias risk assessment as percentages across all RCTs. Bias judgment was based on “The Cochrane Collaboration’s tool for assessing risk of bias in randomized trials”. Supplementary File S3 shows a summary of the risk of bias assessment of each item for each included RCT. 

In all RCTs, the selection process and the randomization process were not clearly described. It was therefore impossible to make a statement about the selection bias in the studies. Two of the 4 studies were blinded. There was a low risk of detection bias since the main outcome in the RCTs was overall survival. There did not seem to be other forms of bias in the selected RCTs.

The ROBINS-I tool, used for the non-randomized comparative studies, showed moderate to high risk of bias for the majority of the comparative studies as seen in Table 2. The Effective Public Health Practice Project tool, used for the non-randomized non-comparative studies, showed moderate to weak quality of the majority of non-comparative studies as seen in Table 3. Although certain studies used broad selection criteria, selection bias is hard to avoid without randomization.

###  3.4. Results of Individual Studies 

Results of the individual studies are summarized in Table 1.

#### 3.4.1. Overall Survival

Ten of the included studies measured overall survival in vitamin C treated patients [1,3,23,24,26,27,29,30,32,37] (Table 1). All studies compared their results with those obtained with a control group. In three studies, no effect of vitamin C on survival time was observed [3,29,32]. Two of those studies were RCTs. In 7 studies, the researchers found a positive effect of vitamin C on survival time [1,23,24,26,27,30,37], two of those studies were RCTs. One of these RCTs was done in acute myeloid leukemia in a small group of patients treated with a hypomethylating agent (decitabine) that in vitro has a synergistic effect on vitamin C on TET2 expression, apoptosis and proliferation of tumor cells. Patients received a relatively low dose of vitamin C intravenous and median overall survival increased with 6 months [37]. In the other RCT, the overall survival also trended toward improvement with vitamin C addition to standard chemotherapy for ovarian cancer, but since the patient groups were very small the increase in median overall survival was not significant [27]. 

##### Intravenous versus Oral Vitamin C

All studies in which researchers suggested a positive effect of vitamin C on survival time, supplementation was administered intravenously, with [1,23,24,30] or without [26,27,37] oral supplementation. In the 3 studies, in which no effect of vitamin C on survival was observed, the supplementation was administered only orally [3,29,32]. 

#### 3.4.2. Clinical Response

The effect of vitamin C on clinical response was assessed in 9 studies [2,25,26,28,29,30,31,33,37], of which six showed at least some clinical improvement [2,25,26,28,30,37]. In the first study in patients with advanced stage various types of cancer, in which some positive effects were described, 10% of the patients experienced tumor regression [2]. This regression, however, was mostly measured based on clinical findings (and not imaging) and earlier tumor progression or metastasis was not always histologically proven. Other clinical improvements observed in that study were also highly subjective. 

When IVC was given in 14 patients with various types of advanced cancer in combination with cytotoxic chemotherapy, 43% of the patients experienced a transient, but sometimes long-lasting stable disease; the investigators thought this was the effect of the vitamin C since it was highly unlikely to be due to chemotherapy alone [25]. However, no tumor regression was seen. In 15 patients with bone metastasis IVC had a positive effect on relief of pain [26]: with IVC 53% of patients had an significant improvement of the pain versus 13% with chemotherapy and 0% in controls without treatment. Another research group also observed a decrease in pain and decreased use of narcotic drugs [30]. They also saw some subjective other clinical improvements possibly related to the treatment with vitamin C. 

In 75% of 20 prostatic cancer patients, PSA (prostate-specific antigen) decreased after the administration of IVC [28]. This effect, however, was not confirmed in another trial were none out of 23 patients with prostate cancer experienced a reduction of the PSA [31].

In 2 other studies there was also no objective clinical improvement [29,33]. In one of these (an RCT) 64% of vitamin C patients claimed relief of pre-treatment symptoms, but this was similar in the placebo group (65%) [29]. 

The only objective improvement of clinical response was seen in patients with acute leukemia; patients with vitamin C had significantly better complete response rates after chemotherapy than without [37].

##### Intravenous Versus Oral Vitamin C

All investigators that reported positive effects of vitamin C on clinical response administered vitamin C IV, with [2] or without [25,26,28,37] oral vitamin C. 

#### 3.4.3. Quality of Life and Performance Status

QOL and/or PS after vitamin C treatment were assessed in 7 of the included studies [3,26,30,31,34,35,36]. In 5 studies, investigators showed a beneficial effect of vitamin C [26,30,34,35,36] while in 2 studies no benefit of vitamin C could be demonstrated [3,31]. 

Remarkable is one RCT in which 63% of the 60 patients with various types of advanced stage cancer on vitamin C claimed some improvement in symptoms but also 58% of the patients on placebo [3].

##### Intravenous Versus Oral Vitamin C

Vitamin C was given IV (+/- orally) in the studies in which the investigators demonstrated a beneficial effect of vitamin C on QOL and PS [26,30,34,35,36]. In 1 study in which no effect was seen vitamin C was given intravenously in combination with a low dose of vitamin C orally, in the other study without benefit only oral supplementation was used [3,31]. 

#### 3.4.4. Safety and Toxicity

Fourteen articles report on the safety and toxicity of vitamin C treatment [2,3,22,25,26,27,29,30,31,33,34,35,36,37]. In all studies, no vitamin C related toxicity was observed. In 10 studies, possible side effects of vitamin C were seen but these were generally mild, and in general not more than in a control group. In one of these studies in which vitamin C was given through continuous infusions, the investigators described 2 serious adverse events (SAEs) that were possibly related to vitamin C treatment: kidney stones and hypokalemia [33]. In 2 studies, no vitamin C related side effects were observed [27,35]. Investigators of 2 studies only described that there were no SAEs [30,36]. 

#### 3.4.5. Vitamin C Concentrations after Supplementation

In 8 of the studies, plasma or serum concentrations of vitamin C were measured [24,25,27,28,31,32,33,34]. In 4 of those studies, baseline values were mentioned by the authors [25,31,32,33]. In 3 of these studies, the plasma vitamin C concentrations before supplementation were normal to high, ranging between 45 to 66 µM on average. In 14 of 22 patients with late-stage terminal cancer, the investigators describe low mean baseline values, but write down that the mean plasma value was 100 µM before supplementation [33]. 

All 8 studies mentioned plasma or serum vitamin C concentrations after the start of supplementation. Two studies dosed vitamin C in participants based on peak plasma concentrations after IVC with a target range of 20–23 mM. In 13 patients with ovarian cancer, it is not mentioned if this goal was always reached; in 60 patients with various types of newly diagnosed cancer only 54% of patients reached the required level at the end of the treatment period. Three other articles also describe peak plasma levels directly after IVC between 14 to 19.3 mM [25,28,31].

In 3 studies it is not explained at what moment in time plasma vitamin C concentrations were measured after supplementation. In the study in which mostly vitamin C deficient patients were treated, mean plasma levels during IVC were 1.1 mM (range 0.38–3.0) [33]. In terminal cancer patients, the majority of the 1532 control patients had plasma concentrations of less than 85 µM, while the majority of the 294 vitamin C supplemented patients had concentrations higher than 114 µM [24]. In newly diagnosed breast cancer patients, researchers used oral supplementation in patients with normal baseline plasma vitamin C concentrations and measured values between 111 to 124 µM after supplementation [32]. 

In 2 studies it is mentioned that leukocyte vitamin C levels were measured [24,32], but in only 1 study the results are given. In that study, the investigators did not see a significant difference in leukocyte vitamin C concentrations in 27 patients with newly diagnosed breast cancer before and after the start of oral vitamin C supplementation.

## 4. Discussion

### 4.1. Summary of the Main Results

This systematic review presents the results and quality assessment of 19 studies to evaluate the effect of vitamin C treatment in cancer patients. It is difficult to draw any conclusions, since these studies have a large variety of outcome measures and the included studies also differ in study population (from newly diagnosed to advanced cancer), co-interventions (none or various adjuvant anti-cancer treatments), and vitamin C treatment (different doses, schedules and modes of delivery). The results of some studies do suggest that vitamin C might have a positive effect on overall survival, clinical response, QOL and PS. However, this effect cannot be generalized to all patient groups with cancer. The best indication of a positive effect of vitamin C is seen in the RCT where it was used intravenously in elderly acute myeloid leukemia patients who were also treated with decitabine. This effect is most likely due to direct regulation of ten-eleven-translocation (TET) activity by vitamin C in synergy with decitabine. TET enzymes are dioxygenases that are important for DNA methylation and are often less functional in patients with AML. One of the potential working mechanisms of decitabine already is the upregulation of the TET proteins; it is thought that vitamin C enhances this effect [37]. In all other studies, especially those that included patients with various types of cancer, the results were less clear. Therefore, it is not proven and seems quite unlikely that the pro-oxidant capacity of vitamin C in high dosage creates a positive effect on overall survival, clinical response, QOL or PS in cancer patients in general.

More than half of the studies that researched QOL or PS saw a beneficial effect. However, since these studies were not blinded, patients may have experienced the well-known ‘placebo effect’. In this respect, the observation of Creagan et al. is important, since he demonstrates a positive effect in nearly 60% of the patients on placebo. These factors might have favored a positive outcome of vitamin C treatment in all studies, especially those in which outcome was assessed through self-administered questionnaires that are highly subjective. 

The mode of delivery seems to be an important factor in the effectiveness of the vitamin C treatment. In the studies with positive effects intravenous delivery was used, while the absence of effect was mostly after oral administration. This suggests that the vitamin C levels that can be reached by oral supplementation might not be high enough for a possible effect, or that vitamin C is not absorbed from the gastrointestinal tract. 

In the 14 studies in which investigators reported on side effects of vitamin C, these side effects were mostly mild or none at all and could have been related to the cancer itself or to the concomitant therapy patients received. There was no obvious difference in side effects of oral vitamin C compared to intravenous supplementation.

If measured at baseline, only 1 of the studied patient groups was vitamin C deficient. Perhaps supplementation would be more efficient and useful in patient groups that indeed are deficient. Unfortunately, also not many vitamin C plasma concentrations after supplementation were documented, but with oral supplementation plasma concentrations were much lower than with IVC.

### 4.2. Quality of the Evidence 

The quality of the evidence is poor due to the lack of extensive double-blinded RCTs. There are only 4 small RCTs that have been undertaken on this subject, and in only 2 of these RCTs intravenous supplementation of vitamin C was used, which seems to be the most optimal to increase vitamin C concentrations in the patients. 

All articles showed one or more forms of bias in the study procedures. Since only 4 studies were randomized, there is a high risk for selection bias. Especially the absence of blinding procedures has resulted in performance and detection bias. Outcome assessors might have overestimated the effects of vitamin C in patients, based on knowledge of their characteristics and clinical status. 

In general, most studies examined in this review show some positive effects of vitamin C supplementation on cancer. However, this could also be due to a publication bias of studies with a good result and are at best indicative that it is unlikely that vitamin C supplementation in any form is harmful in patients with cancer. 

### 4.3. Potential Biases in the Review Process

Article selection and data extraction was peer reviewed by a second author. Assessment of the quality of included studies was performed independently by two authors, with discrepancies resolved by discussion and a third author when necessary. The authors therefore believe the review process was unbiased. The only limitation is the quality of the included studies.

## 5. Conclusions

The results of this review do not prove that there is a clinically relevant positive effect of vitamin C supplementation in most cancer patients on the overall survival, clinical status, QOL and PS. The quality of the studies published is low and the interventions and patient groups are very diverse. The best indication of a positive effect is seen in acute myeloid leukemia patients in combination with decitabine, and in vitro data also show a synergistic efficacy of both treatments. An effect in some other patient groups might still be possible and might have been overlooked. In addition, clear pharmacological data might be needed to optimize treatment plans. 

Treatment with vitamin C is likely to be safe, with almost no serious adverse events and minimal mild side effects, even with high doses of intravenous supplementation. There are also no indications that cancer progresses faster under vitamin C supplementation. 

We see the results of this review as an indication that it is safe to examine vitamin C supplementation further in a randomized controlled setting. Therefore, we are planning to investigate the effect of vitamin C supplementation on immune recovery in patients that receive intensive chemotherapy and/or stem cell transplantation.

## Figures and Tables

**Table 1:** Characteristics and outcome of the included studies in alphabetical order.

Study	N(Vitamin C/ controls)	Type	Participants	Interven-tion	Concomitant therapy	Main endpoints	Adverse events
Bazzan 2018 [22]	86 (86/0)	Retrospective cohort	All types of cancer in different settings	50 - 150 g IV, at least 5 times (total 3034 doses)	32 patients none, 54 patients chemotherapy	20 of 40 patients improvement of fatigue, 15 of 86 patients improvement of appetite.	Mostly mild adverse events (AEs), like nausea, vomiting and discomfort at injection side (<3% of infusions). Self- limiting to time of infusions. No serious AEs (SAEs) related to vitamin C
Cameron 1974 [2]	50(50/0)	Retrospective cohort	Advanced stage cancer patients	10 g a day IV for 10 days + oral vitamin C	None	10 minimal response, 11 growth retardation, 3 stable disease, 5 tumor regression. Less pain, reduction in ascites/pleural effusions.	Fluid retention, edema, dyspeptic symptoms, tumor hemorrhage/necrosis.
Cameron 1976 [1]	1100(100/1000)	Case-control	Incurable cancer patients	10 g a day IV for 10 days + oral vitamin C	Conventional anti-cancer treatment	Mean overall survival (OS) 210 days vs. 50 days in controls (4.2x more)	Not reported (NR)
Cameron 1978 [23]	1100(100/1000)	Case-control	Terminal cancer patients	10 g a day IV for 10 days + oral vitamin C	None	Recalculation of Cameron 1976. Average OS (7.7x more = 288 days)	NR
Cameron 1991 [24]	1826(294/1532)	Case-control	Terminal cancer patients	10 g a day IV for 10 days + oral vitamin C	None	OS 343 days vs. 180 days in controls	NR
Creagan 1979 [3]	123(60/63)	RCT	Advanced stage cancer patients	10 g a day orally	NR	Identical survival.Performance status (PS) identical. 58% vs. 63% some improvement in symptoms	Nausea, vomiting
Hoffer 2015 [25]	14 (14/0)	Uncontrolled phase II	Advanced stage cancer patients	1.5g/kg IV 2-3 times per week.	Chemothera-py	6 transient, partly long-lasting stable disease	Edema, thirst, nausea, vomiting, headache, chills
Günes-Bayir 2015 [26]	39 (15/24)	Case-control	Bone metastases from various types of cancer	2.5 g IV a day	NR	OS 10 months vs. 2 months in controls. Decrease in pain in 9/15 vs. 5/24 in controls. PS improvement in 4/15 vs. 1/24 in controls.	40% mild diarrhea, 30% mild oliguria
Ma 2014 [27]	25 (13/12)	RCT	Newly diagnosed stage III and IV ovarian cancer after debulking	IV two times per week using a dose escalating protocol (final dose either 75 or 100g) for 12 months.	Paclitaxel and carboplatin chemotherapy	Trend in improvement OS, 25.5 months vs. 16.75 months in controls, (not significant).	Fewer chemotherapy related side effects with vitamin C, no relevant AEs of vitamin C.
Mikirova 2012 [28]	45 (45/0)	Retrospective cohort	Various types of cancer, mostly metastatic	Escalate to 50 g IV 3 times per week for a median of 9 times	NR	76% reduction in C –reactive protein, 75% reduction in prostate-specific antigen (PSA)	NR
Moertel 1985 [29]	100 (51/49)	RCT	Advanced colorectal cancer	10 g a day orally	None	Median OS 2.9 months vs. 4.1 months in controls. 7/11 symptom relief vs. 11/17 in controls	Low incidence of AEs, mild.
Murata 1982 [30]	130 (111/19)	Non randomized clinical trial	Terminal cancer patients	Site 1: 6–30 g a day orally and 10–20g IV. Site 2: 0.5–3 g or 5–30 g per day orally.	NR	Site 1: average OS with high dose vitamin C 246 days vs. 43 days with low dose.Site 2: average OS with high dose vitamin C 115 days vs. 48 days in controls. Less use of narcotic drugs in vitamin C treated patients: 17% in high dose vs. 50% in low dose vs. 79% in controls. Improved state of wellbeing, improved appetite, increased mental alertness	No SAEs
Nielsen 2017 [31]	23 (23/0)	Uncontrolled phase II	Chemothe-rapy-naïve metastatic castration-resistant prostate cancer	Weekly infusions for 12 weeks. Week 1: 5 g, week2: 30 g, week 3–12: 60 g + oral 500 mg/day.	None	75% of patients PSA increase at 12 weeks, one PSA decrease of 27%. 80% unchanged PS at week 12, 2 improved, 2 worse score.Quality of life (QOL) identical baseline to week 12.	53 AE, mostly mild and not related to vitamin C. 11 SAEs, explained by progression of prostate cancer. 2 pulmonary embolisms.
Poulter 1984 [32]	66 (27/25)	Non randomized clinical trial	Newly diagnosed breast cancer	3 g a day orally	NR	No change in survival rates	NR
Riordan 2005 [33]	24 (24/0)	Uncontrolled phase II	Late stage terminal cancer, mostly colorectal (19)	150 to 710 mg/kg/day IV for 8 weeks	None	1 patient stable disease, all others progressive disease.	Mild: nausea (46%), edema (29%), dry mouth or skin (29%), fatigue (25%). Serious: 1 kidney stones, 1 hypokalemia.
Takaha-shi 2012 [34]	60 (60/0)	Prospective cohort	Newly diagnosed cancer of various types	IV dose of 12.5–15 g, 25 g and 50 g + vitamin C orally 2–4 g a day.	Chemothera-py (n = 33), radiation therapy (n = 1), none (n = 2)	Improvement in QOL: score 44.6 before treatment vs. 53.2 at 2 weeks and 61.4 at 4 weeks.	Mild (grade 1), most often headache (8.3%), nausea (8.3%)
Voll-bracht 2011 [35]	125 (53/72)	Retrospective cohort	Breast cancer stages IIa–IIIb.	IV 7.5 g once a week for at least 4 weeks	Primary surgical treatment +/- adjuvant chemotherapy +/- adjuvant radiotherapy	Reduced QOL related side effects, slight increase PS during adjuvant treatment (80% vs. 71%) and aftercare (87% vs. 78%).	None
Yeom 2007 [36]	39 (39/0)	Uncontrolled phase II	Terminal cancer of various types, stage IV.	10 g IV twice + 4 g oral vitamin C daily for a week.	NR	Health score improved from 3637 to 5537 after vitamin C.	No vitamin C supplementation stopped because of side effects.
Zhao 2017 [37]	73 (39/34)	RCT	Newly diagnosis elderly with acute myeloid leukemia	50–80 mg/kg IV during 10 days/month, at most 10 months	Decitabine, cytarabine and aclarubicin chemotherapy	Median OS 15.3 months vs. 9.3 months in controls (p = 0.039). Complete remission rate higher with Vitamin C: 84.6% vs. 70.6% after 2 courses.	Identical amount of AEs and SAEs in both groups.

**Table 2:** Risk-of –bias assessment of comparative studies. Judgment for risk of bias based on the ROBINS-I tool for each included comparative study (high, moderate, low).

Study	Evidence of Selection Bias/Prognostic Imbalance	Bias Due to Confounding Factors	Bias in Measurement of Outcomes	Follow-up of Participants Sufficiently Complete	Bias Due to Selection of Reported Results or due to Missing Data	Comparability of Cohorts on Important Confounding Factors
Cameron 1976 [1]	High risk	High risk	Moderate risk	Moderate risk	Low risk	Moderate risk
Cameron 1978 [23]	High risk	High risk	Moderate risk	Moderate risk	Low risk	Moderate risk
Cameron 1991 [24]	Moderate risk	High risk	Low risk	Moderate risk	Low risk	Moderate risk
Günes-Bayir 2015 [26]	Moderate risk	Moderate risk	Moderate risk	Moderate risk	Low risk	Moderate risk
Murata 1982 [30]	High risk	High risk	Moderate risk	Moderate risk	Moderate risk	Moderate risk
Poulter 1984 [32]	High risk	Moderate risk	Moderate risk	High risk	Moderate risk	Moderate risk
Vollbracht 2011 [35]	High risk	Moderate risk	Moderate risk	Low risk	Low risk	High risk

**Table 3:** Quality assessment of non-comparative studies. Judgment for quality of the studies based on the Effective Public Health Practice Project tool for each included non-comparative study (strong, moderate, weak).

Study	Selection	Study Design	Confounders	Blinding	Data Collection Methods	Withdrawals and Drop-Outs
Bazzan 2018 [22]	Weak	Weak	Weak	Weak	Weak	Not applicable
Cameron 1974 [2]	Moderate	Weak	Weak	Weak	Moderate	Not applicable
Hoffer 2015 [25]	Moderate	Weak	Weak	Weak	Strong	Strong
Mikirova 2012 [28]	Weak	Weak	Weak	Weak	Moderate	Strong
Nielsen 2017 [31]	Moderate	Weak	Moderate	Weak	Strong	Strong
Riordan 2005 [33]	Moderate	Weak	Weak	Weak	Strong	Strong
Takahashi 2012 [34]	Moderate	Weak	Weak	Weak	Moderate	Strong
Yeom 2007 [36]	Moderate	Weak	Weak	Weak	Moderate	Strong

## Supplementary Materials

The following are available online at https://www.mdpi.com/2072-6643/11/5/977/s1, Supplementary File S1: Literature Search, Supplementary File S2: Used risk of bias tools, Supplementary File S3: Risk of bias summary.
