# Supplementary material for: The Effect of Vitamin C (Ascorbic Acid) in the Treatment of Patients with Cancer: A Systematic Review
Source: Nutrients. 2019 Apr 28;11(5):977. doi: 10.3390/nu11050977 (PMC6566697; doi:10.3390/nu11050977)
Supplement: Supplementary file 1 [file nutrients-11-00977-s001.pdf]

Supplemental Materials

Supplemental Material for:
The effect of vitamin C (ascorbic acid) in the treatment of patients with cancer: a systematic review.

G. van Gorkom, E. Lookermans, C. van Elssen, G. Bos.

Supplemental File S1: Literature Search

Database Pubmed:

Search Strategy:

((((((Cancer) OR Neoplasm) OR "Neoplasms"[Mesh])) AND ((((((dehydroascorbic acid) OR Ascorbate) OR Vitamin C) OR ascorbic acid) OR "Dehydroascorbic Acid"[Mesh]) OR "Ascorbic Acid"[Mesh])) AND (((((((((((Randomized) OR RCT) OR randomized controlled trial) OR "Randomized Controlled Trials as Topic"[Mesh]) OR "Randomized Controlled Trial"[Publication Type])) OR ((((Clinical trial) OR Controlled clinical trial) OR "Controlled Clinical Trials as Topic"[Mesh]) OR "Controlled Clinical Trial"[Publication Type])) OR ((Case control) OR "Case-Control Studies"[Mesh])) OR ((Prospective Study) OR "Prospective Studies"[Mesh])) OR (((cohort) OR cohort study) OR "Cohort Studies"[Mesh])) OR (((Phase 2) OR "Clinical Trial, Phase II"[Publication Type]) OR "Clinical Trials, Phase II as Topic"[Mesh])) OR (((Observational study) OR "Observational Study"[Publication Type]) OR "Observational Studies as Topic"[Mesh]))) AND ((((((((Response rate) OR Tumor response) OR Toxicity) OR progression free survival) OR Overall survival) OR Reduced infection) OR ((Disease free survival) OR "Disease-Free Survival"[Mesh])) OR ((Quality of life) OR "Quality of Life"[Mesh]))
Filter: Human, English.

Database EMBASE:
Search strategy: ((vitamin C) OR (ascorbic acid) OR ascorbate) AND (neoplasm* OR malignanc* OR cancer OR carcinoma OR leukemia OR lymphoma)

Filter: Human, English.

Supplemental File S2: Used risk of bias tools

Cochrane Collaboration's tool for assessing risk of bias

**Bias domain**	**Source of bias**	**Support for judgment**	**Review authors' judgment (assess as low, unclear or high risk of bias)**
Selection bias	Random sequence generation	Describe the method used to generate the allocation sequence in sufficient detail to allow an assessment of whether it should produce comparable groups	Selection bias (biased allocation to interventions) due to inadequate generation of a randomised sequence
	Allocation concealment	Describe the method used to conceal the allocation sequence in sufficient detail to determine whether intervention allocations could have been foreseen before or during enrolment	Selection bias (biased allocation to interventions) due to inadequate concealment of allocations before assignment
Performance bias	Blinding of participants and personnel	Describe all measures used, if any, to blind trial participants and researchers from knowledge of which intervention a participant received. Provide any information relating to whether the intended blinding was effective	Performance bias due to knowledge of the allocated interventions by participants and personnel during the study
Detection bias	Blinding of outcome assessment	Describe all measures used, if any, to blind outcome assessment from knowledge of which intervention a participant received. Provide any information relating to whether the intended blinding was effective	Detection bias due to knowledge of the allocated interventions by outcome assessment
Attrition bias	Incomplete outcome data	Describe the completeness of outcome data for each main outcome, including attrition and exclusions from the analysis. State whether attrition and exclusions were reported, the numbers in each intervention group (compared with total randomised participants), reasons for attrition or exclusions where reported, and any reinclusions in analyses for the review	Attrition bias due to amount, nature, or handling of incomplete outcome data
Reporting bias	Selective reporting	State how selective outcome reporting was examined and what was found	Reporting bias due to selective outcome reporting
Other bias	Anything else, ideally prespecified	State any important concerns about bias not covered in the other domains in the tool	Bias due to problems not covered elsewhere

ROBIN-1 tool

Domain	Explanation
**Pre-intervention**	**Risk of bias assessment is mainly distinct from assessments of randomised trials**
Bias due to confounding	Baseline confounding occurs when one or more prognostic variables (factors that predict the outcome of interest) also predicts the intervention received at baseline<br>ROBINS-I can also address time-varying confounding, which occurs when individuals switch between the interventions being compared and when post-baseline prognostic factors affect the intervention received after baseline
Bias in selection of participants into the study	When exclusion of some eligible participants, or the initial follow-up time of some participants, or some outcome events is related to both intervention and outcome, there will be an association between interventions and outcome even if the effects of the interventions are identical<br>This form of selection bias is distinct from confounding—A specific example is bias due to the inclusion of prevalent users, rather than new users, of an intervention
**At intervention**	**Risk of bias assessment is mainly distinct from assessments of randomised trials**
Bias in classification of interventions	Bias introduced by either differential or non-differential misclassification of intervention status<br>Non-differential misclassification is unrelated to the outcome and will usually bias the estimated effect of intervention towards the null<br>Differential misclassification occurs when misclassification of intervention status is related to the outcome or the risk of the outcome, and is likely to lead to bias
**Post-intervention**	**Risk of bias assessment has substantial overlap with assessments of randomised trials**
Bias due to deviations from intended interventions	Bias that arises when there are systematic differences between experimental intervention and comparator groups in the care provided, which represent a deviation from the intended intervention(s)<br>Assessment of bias in this domain will depend on the type of effect of interest (either the effect of assignment to intervention or the effect of starting and adhering to intervention).
Bias due to missing data	Bias that arises when later follow-up is missing for individuals initially included and followed (such as differential loss to follow-up that is affected by prognostic factors); bias due to exclusion of individuals with missing information about intervention status or other variables such as confounders
Bias in measurement of outcomes	Bias introduced by either differential or non-differential errors in measurement of outcome data. Such bias can arise when outcome assessors are aware of intervention status, if different methods are used to assess outcomes in different intervention groups, or if measurement errors are related to intervention status or effects
Bias in selection of the reported result	Selective reporting of results in a way that depends on the findings and prevents the estimate from being included in a meta-analysis (or other synthesis)

Effective Public Health Practice Project (EPHPP) Quality Assesment Tool for Quantitative Studies

Component ratings

A. Selection bias
    1. Are the individuals selected to participate in the study likely to be representive of the target population?
        a. Very likely
        b. Somewhat likely
        c. Not likely
        d. Can't tell
    2. What percentage of selected individuals agreed to participate?
        a. 80-100% agreement
        b. 60-79% agreement
        c. less than 60% agreement
        d. Not applicable
        e. Can't tell

B. Study Design

   Indicate study design
    1. Randomized controlled trial
    2. Controlled clinical trial
    3. Cohort analytic (two group pre + post)
    4. Case-control
    5. Cohort (one group pre + post (before and after))
    6. Interrupted time series
    7. Other specify...
    8. Can't tell

   Was the study describes as randomized? If No go to component C.
   If Yes, was the method of randomisation described?
   If Yes, was the method approproate?

C. Confounders
    1. Were there important differences between groups prior to intervention?
        a. Yes
        b. No
        c. Can't tell
    2. If yes, indicate the percentage of relevant confounders that were controlled
        a. 80-100% (most)
        b. 60-79% (some)
        c. Less than 60% (few or none)
        d. Can't tell

D. Blinding
    1. Was (were) the otcome assessor(s) aware of the intervention or exposure status of participants?
        a. Yes
        b. No
        c. Can't tell
    2. Were the study participants aware of the research question?
        a. Yes
        b. No
        c. Can't tell

E. Data collection methods
    1. Were data collection tools shown to be valid?

        a. Yes
        b. No
        c. Can’t tell
    2. Were data collection tools shown to be reliable?
        a. Yes
        b. No
        c. Can’t tell

F. Withdrawals and drop-outs
    1. Were withdrawals and drop-outs reported in terms of numbers and/or reasons per group?
        a. Yes
        b. No
        c. Can’t tell
        d. Not applicable
    2. Indicate the percentage of participants completing the study.
        a. 80-100%
        b. 60-79%
        c. less than 60%
        d. Can’t tell
        e. Not applicable

Supplemental File S3: Risk of bias summary

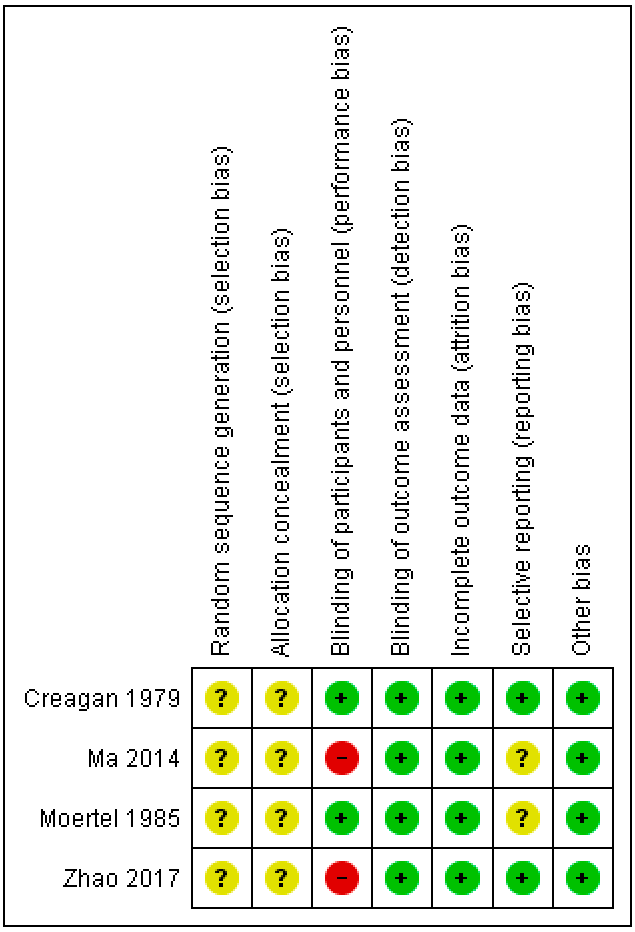

Itemized judgments for risk of bias item for each individual included RCT (+: low risk of bias; ?: unclear risk of bias; -: high risk of bias).
